# Radon prevalence in domestic water in the Ría de Vigo coastal basin (NW Iberian Peninsula)

**DOI:** 10.1007/s11356-023-27305-6

**Published:** 2023-05-04

**Authors:** Juan Severino Pino Ibánhez, Xosé Antón Álvarez-Salgado, Carlos Rocha

**Affiliations:** 1 Laboratorio de Geoquímica Orgánica, Instituto de Investigacións Mariñas (IIM), Consejo Superior de Investigaciones Científicas (CSIC), Vigo, Spain; 2 Biogeochemistry Research Group, School of Natural Sciences, Trinity College Dublin, Dublin, Ireland

**Keywords:** ^222^Rn, Radon risk, Radiation exposure, Domestic water use, Cancer risk, Crystalline aquifer

## Abstract

The Ría de Vigo catchment is situated in the largest radon-prone area of the Iberian Peninsula. High local indoor radon (^222^Rn) levels are the preeminent source of radiation exposure, with negative effects on health. Nevertheless, information on radon levels of natural waters and the potential human exposure risks associated with their domestic use is very sparse. To elucidate the environmental factors increasing human exposure risk to radon during domestic water use, we undertook a survey of local water sources, including springs, rivers, wells, and boreholes, over different temporal scales. Continental waters were highly enriched in ^222^Rn: activities ranged from 1.2 to 20.2 Bq L^−1^ in rivers and levels one to two orders of magnitude higher were found in groundwaters (from 8.0 to 2737 Bq L^−1^; median 121.1 Bq L^−1^). The geology and hydrogeology of local crystalline aquifers support one order of magnitude higher ^222^Rn activities in groundwater stored in deeper fractured rock compared to that contained within the highly weathered regolith at the surface. During the mean dry season, ^222^Rn activities nearly doubled in most sampled waters in comparison to the wet period (from 94.9 during the dry season to 187.3 Bq L^−1^ during wet period; *n* = 37). Seasonal water use and recharge cycles and thermal convection are postulated to explain this variation in radon activities. The high ^222^Rn activities cause the total effective dose of radiation received from domestic use of untreated groundwaters to exceed the recommended 0.1 mSv y^−1^. Since more than 70% of this dose comes from indoor water degassing and subsequent ^222^Rn inhalation, preventative health policy in the form of ^222^Rn remediation and mitigation measures should be implemented prior to pumping untreated groundwater into dwellings, particularly during the dry period.

**Supplementary Information:**

The online version contains supplementary material available at 10.1007/s11356-023-27305-6.

## Introduction

Radon (^222^Rn) is a radioactive noble gas, resulting from the disintegration of ^226^Ra in the ^238^U decay chain. The short half-life of ^222^Rn (3.8 days) means that it is a continuous source of natural radiation. The decay of radon emits an α particle following a chain that also includes the short-lived, α-emitting ^218^Po, ^214^Po, and ^210^Po (e.g., NRC 1999). U and Ra are widely found in rocks and soils and thus, are continuous natural sources of ^222^Rn. Unlike its parent isotope ^226^Ra, which can be found as part of the rock matrix or dissolved in natural water, ^222^Rn is highly mobile once produced. It is often found accumulated in water and enclosed spaces above and below ground, and can reach large concentrations, delivering large doses of radiation to biological tissue that can cause damage to DNA (e.g., Lehnert and Goodwin 1997; Meenakshi et al. 2017).

The ^238^U content of the local bedrock has been shown as a main driver of the distribution of ^222^Rn activities within watersheds (e.g., Banks et al. 1998; Duggal et al. 2020; Sukanya et al. 2021). However, geologic and hydrogeologic drivers can also be determinants of the magnitude and variability of ^222^Rn activities in continental waters. Factors such as chemical weathering, ^226^Ra precipitation and other air–water-rock interactions strongly affect ^222^Rn emanation rates, which in turn affect ^222^Rn levels in continental waters (Girault et al. 2018; Przylibski 2000, 2011). Specific yield, transmissivity, water content, or preferential flow can also drive large spatial gradients in ^222^Rn activities in both groundwater and connected surface waters (e.g., Hoehn and Gunten 1989; Veeger and Ruderman, 1998; Jiang et al. 2021; Sukanya et al. 2021). Temporal variations in ^222^Rn activities in groundwater at the decadal timescale are associated with tectonic and cosmic drivers (Finkelstein et al. 1998; Yan et al. 2017). These overlay daily and seasonal cycles driven by environmental factors such as groundwater recharge-discharge cycles or temperature gradients (Choubey et al. 2011; Kamra 2015). These complex dynamics in the aquatic environment result in variable ^222^Rn activities in continental water resources, and hence human exposure to water-borne radiation.

Exposure to ^222^Rn and its progeny is the largest, single natural source of ionizing radiation to humans (UNSCEAR 1993, 2000). In 1988, the International Agency for Research on Cancer declared ^222^Rn and its progeny human carcinogens based on epidemiological studies of underground uranium miners (IARC 1988). Currently, residential ^222^Rn exposure is considered the second most important cause of lung cancer after smoking (WHO 2009). Due to its ubiquity and pernicious effects on human health, indoor ^222^Rn monitoring and mitigation measures have been implemented worldwide by law (e.g., Council Directive 2013/51/Euratom in the European Union). Although ^222^Rn found in air or water originates primarily from the local geology, both environmental levels and exposure routes differ according to the medium, and consequently, mitigation actions are distinct. Despite epidemiological and physiological evidence of the risks associated with human exposure to ^222^Rn derived from domestic water use (e.g., Yu and Kyu Kim 2004; Messier and Serre 2017), it has received less attention compared to ^222^Rn in air within dwellings both at the epidemiological and legislative level.

This study was conducted in a coastal basin located within the largest ^222^Rn-prone area of the Iberian Peninsula (Barros-Dios et al. 2007; Llerena et al. 2013; López-Abente et al. 2018). This region covers the northwest and center of the peninsula, where some of the highest concentrations of uranium in topsoil (Tollefsen et al. 2016) and indoor ^222^Rn levels in Europe can be found (Elío et al. 2019), because of the predominance of granite basement rocks (Quindós Poncela et al. 2004). Given the increased awareness of the potential health risks of radon exposure, several studies have been conducted in this region (e.g., Barros-Dios et al. 2007; López-Abente et al. 2018; Quindós et al. 2008; Quindós Poncela et al. 2004). Quindós et al. (2008) reported indoor ^222^Rn levels exceeding 200 Bq m^−3^ in 68.5% of the 600 residences sampled in the autonomous region of Galicia. Epidemiological studies have linked this high ^222^Rn-in-air exposure with the local incidence of lung and other cancers (López-Abente et al. 2018; Ruano-Ravina et al. 2017; Ruano-Ravina et al. 2021). However, limited information is available on the ^222^Rn content of natural waters in the region and the associated health risk arising from radiation exposure during domestic water use (Llerena et al. 2013 and references therein).

The environmental factors controlling ^222^Rn activity levels in different water sources of Galicia, as well as the associated human health risks, are largely unknown. Filling this knowledge gap is particularly urgent since groundwater meets a significant portion of the local population water demand. Our study aims to evaluate whether the domestic use of continental waters of the Ría de Vigo basin represents a potential harmful ionizing radiation source to the population. Sampling throughout the basin during the wet and dry seasons was carried out to evaluate the influence of water use, local geology, and meteorological conditions over ^222^Rn levels in rivers, wells, boreholes, and springs. Seasonal to weekly variability in ^222^Rn levels is evaluated in selected water supply points to explore the potential factors affecting temporal radon risk variability in the study area, and facilitate and inform effective remediation and mitigation policies. Radiation dosage associated with ^222^Rn ingestion, as well as inhalation of radon derived from indoor water degassing is calculated from our results. The total exposure risk to humans resulting from the domestic use of the local natural waters is then calculated.

## Methods

### Study area

**Fig. 1:**
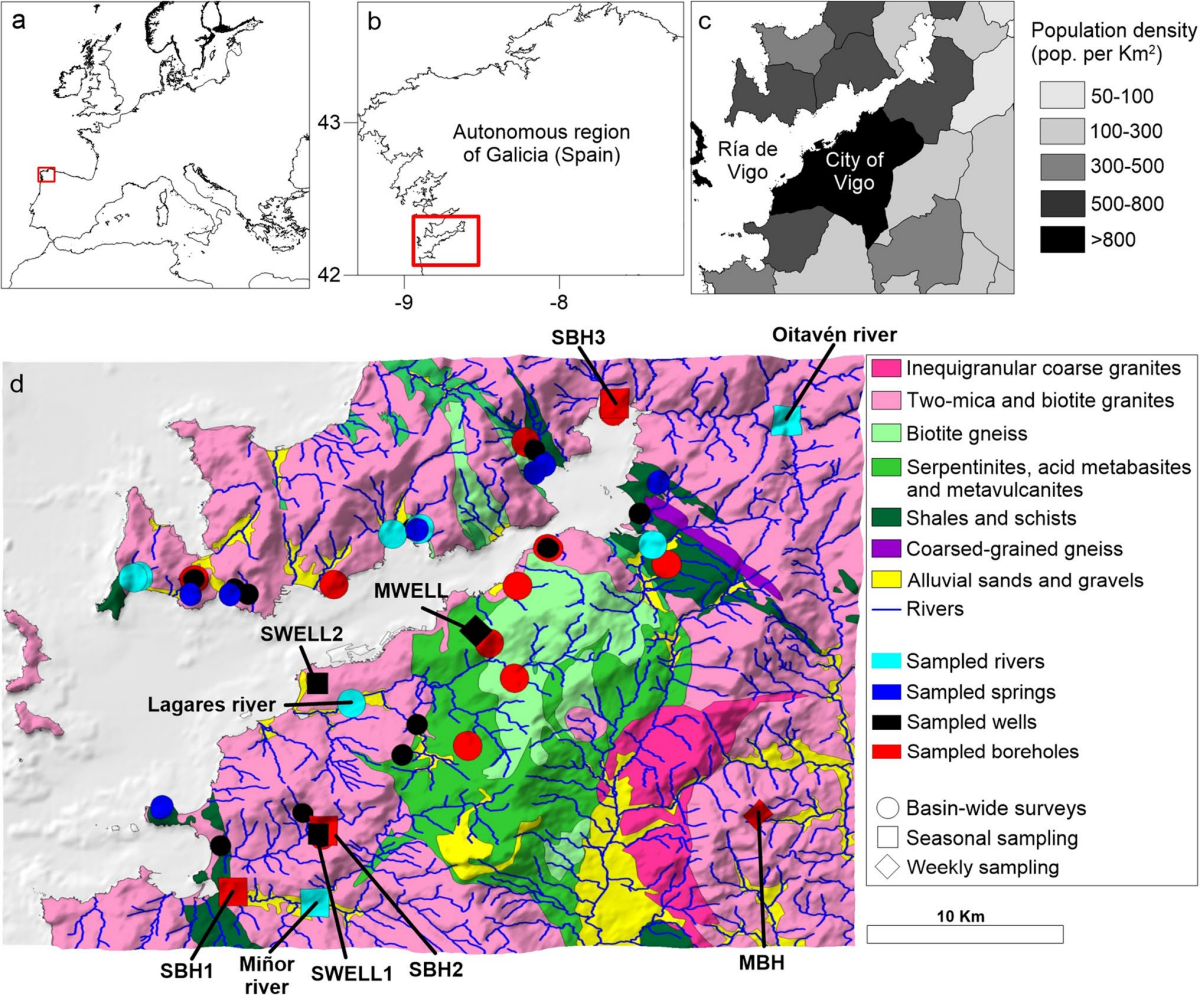
Location of the Ría de Vigo basin (NW Iberian Peninsula, **a**, within the autonomous region of Galicia (Spain; **b** population density, **c** geological composition, **d** and hydrographic network are shown, together with the location of the sampled rivers, wells, springs, and boreholes. Note that different symbols denote the different sampling strategies followed by the study. The codes identifying wells and boreholes sampled seasonally and weekly (see below) are also shown. Population density data was retrieved from the official Spanish 2019 census (Instituto Nacional de Estadística; www.ine.es). Geological information is adapted from IGME (2003)

The Ría de Vigo is a large (176 km^2^), semi enclosed embayment in the northwestern coast of the Iberian Peninsula (Fig. 1) encroached into a drainage basin of 709 km^2^. The climate in the region is temperate, with average temperatures of ~ 15 °C, although there is a significant gradient in air temperature, evapotranspiration, and precipitation from the coast to the mountainous inland (see supplementary materials). Precipitation is concentrated mainly in autumn and winter and can vary from ~ 900 mm in the western shores to ~ 2500 mm in the easternmost part of the basin.

The recent large increase in population in the study area rely on a flourishing economy, initially supported by the high productivity of this coastal system, driven by the seasonal upwelling of nutrient-rich ocean waters. The city of Vigo (population density > 2700 inhabitants per km^2^ in 2019; Fig. 1), along with other districts in the basin, experienced a six-fold increase in population during the twentieth century (Fernández et al. 2016). The fast population growth extended the main residential centers to proximity with rural areas. The major local rivers (the Verdugo-Oitavén River system and the Miñor River; Fig. 1) are the main water sources supplying these populations. Beyond them, and due to the characteristically large dispersion of inhabitants throughout the area, the water supply relies mostly on public and private groundwater extraction. About a quarter of the total regional population is dependent on groundwater (Romay and Gañete 2007).

### Geological and hydrogeological setting

The geology of the Ría de Vigo basin is dominated by a crystalline basement, consisting mostly of Paleozoic alkaline and calc-alkaline granitic and metamorphic rocks (shales, schists, and gneisses) that usually reach the soil surface (Fig. 1). Quaternary deposits associated with alluvial transport are also sporadically found (Fig. 1). These crystalline rocks are characterized by low permeability, and strong horizontal stratification caused mainly by different weathering stages across the study area (Raposo et al. 2012; Naves et al. 2021).

**Fig. 2:**
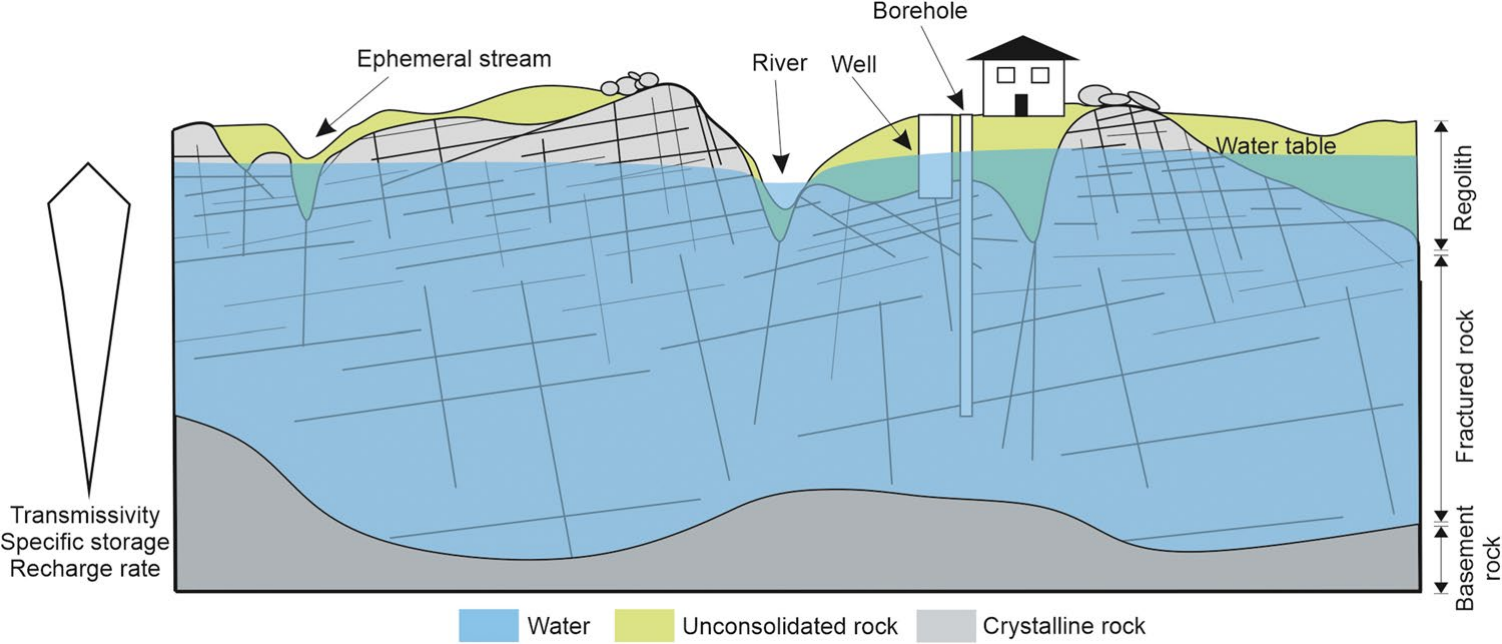
Scheme of the vertical zonation of the crystalline aquifers found in the study area. Typical depth of screening of dug wells and boreholes is represented schematically. Not to scale

The local aquifers are configured in a two-layered system based on the dominant weathering zonation: the upper aquifer is contained within the highly weathered regolith that presents variable thickness typically from 5 to 20 m depth (Fig. 2; Raposo et al. 2013). These upper parts commonly show large horizontal flow transfer following the local topography (e.g., Roques et al. 2014; Calvo-Martin et al. 2021) and provide the majority of the groundwater storage capacity (Soriano and Samper 2000; Raposo et al. 2012; Naves et al. 2021). This determines strong phreatic level fluctuations associated with short groundwater residence times, and storage highly dependent on rainfall recharge. Underneath, the thickness of the fractured rock aquifer is also highly variable, up to 100 m, but frequently reaches the surface. The storage capacity and groundwater flow in this unit are two orders of magnitude lower than that of the upper aquifer (Raposo et al. 2012 and references therein), and dependent on the magnitude and direction of the fractures (Naves et al. 2021). Below these two layers, fractures disappear progressively and thus the fresh basement rock is considered essentially impermeable (Fig. 2).

### Sampling strategy

Two surveys were carried out across the basin in 2018. The first survey took place towards the end of the period of concentrated rainfall (February to May, hereafter the winter/spring period), while the second survey was before the fall rain season began (September to October, hereafter the summer/autumn period). Water samples were collected from 8 rivers, 7 springs, 16 wells, and 14 boreholes throughout the basin (Fig. 1). Groundwater supply points dug by hand into the regolith, with depths typically not exceeding 15 m are defined as wells. Boreholes, on the other hand, are drilled into the fractured rock with screening depths exceeding 20 m (up to 120 m in those included in our sample). Therefore, wells supply groundwater sourced largely from the surface aquifer unit, while boreholes supply groundwater from the fractured rock aquifer unit underneath. River water samples were collected from the subsurface (0.5 m depth) using a Niskin water sampler and transferred to air-tight glass bottles (250 mL) for subsequent analysis. Well and borehole water samples were collected from the tap nearest to the source. Water was pumped until a constant temperature was reached at the outflow to ensure complete purging of the water stagnating in pipes (typically 10–15 min). Spring water samples were collected directly from the outflow ensuring no degassing of the samples occurred. During the 2018 winter/spring survey, springs were underrepresented (*n* = 2). Thus, additional spring samples (*n* = 5) were collected during the winter/spring period (May) in 2021 (see supplementary materials for a comparison of air temperature, rainfall, and water balance for February to April between 2018 and 2021). All water samples for ^222^Rn analysis were collected in 250 mL air-tight glass bottles.

For seasonal monitoring of ^222^Rn levels, 2 rivers, 2 wells, and 3 boreholes were selected (Fig. 1). Water samples were collected quarterly from February 2018 to January 2019. Additionally, two monitoring sites, one well (14 m depth) and one semi-public water supply outlet, supplied mainly from a borehole screened at 120 m, with minor contributions from two nearby springs, were sampled on a weekly basis from March to December 2019 (Fig. 1). The local semi-public system pumps water from the borehole and the springs into a storage tank of 50 m^3^ before distributing to about 40 dwellings, while the sampled well is the main water source for a private house. Although the borehole is located outside the Ría de Vigo basin, it is screened within the same prevalent geological unit (two mica granites; Fig. 1) and thus, taken to be representative of the short-term ^222^Rn variability in boreholes of the study area. Sampling followed the same procedures adopted for the basin-wide surveys.

### Determination of ^222^Rn and ^226^Ra in water samples

A Durridge RAD7 Radon detector with a RadH2O accessory (Durridge Company, Inc., USA) was used to determine ^222^Rn in water. All samples were analyzed within 2 days of collection. Results were corrected for internal ^222^Rn decay from the time of collection to analysis. Due to the high ^222^Rn levels found in most groundwater samples, pre-dilution with ^222^Rn-free water was performed prior to analysis. Known sample aliquots were transferred into the bottom of 250-mL air-tight glass bottles prefilled with ^222^Rn-free water to avoid degassing of the aliquot. Between measurements, the measuring loop was purged through an activated carbon ^222^Rn trap until zero ^222^Rn activities were read.

Additional water samples were collected for the determination of ^226^Ra activity during the summer/autumn basin-wide (dry season) survey. Two treatments were used on replicate samples; both groups were first purged with ^222^Rn-free air and one of them was subsequently filtered through GF/F filters (Whatman, 0.7 μm average pore size) to remove particles. Filtered and unfiltered samples were then stored in 250-mL gas-tight bottles for at least 30 days (i.e., more than 5 times the ^222^Rn half-life) to allow secular equilibration between ^222^Rn and ^226^Ra. The ^226^Ra activity was then determined based on the ingrowth of ^222^Rn activity during storage.

### Domestic ^222^Rn degassing from shower systems

To illustrate the effect of residential groundwater use on domestic ^222^Rn-in-air levels, a private bathroom (1.4 × 2.6 m), equipped with a shower using the semi-public water supply system monitored on a weekly basis in 2019 was repurposed into a large degassing chamber. Water was allowed to flow through the shower head for different periods of time (5, 10, and 15 min) at a constant water flux (7.3 L min^−1^) while air inside the bathroom was pumped through the sampling loop of a RAD7 radon monitor recording for 5-min cycles during a total of 85 min. Radon background levels inside the bathroom were measured for 10 min prior to the shower being turned on. Temperature of water flowing through the shower was 16.0 °C.

### Ancillary data

A high-resolution terrain model of the study area was used to obtain the altitude of the sampling points and the river catchment areas. Climatological data (air temperature, rainfall, water balance) during the period covered by this study (2018–2021) was obtained from the public network of meteorological stations (13 stations in the study area) managed by MeteoGalicia (https://www.meteogalicia.gal). The discharge of the basins’ main rivers was obtained from the public gauging station network managed by Augas de Galicia (https://augasdegalicia.xunta.gal).

Water usage is qualitatively classified for the purposes of this study into three categories: (a) regular, when the water supply is used in the household in a volumetrically sustained way throughout the year; (b) irrigation, when groundwater supply is used for both household activities and irrigation, i.e., water consumption increases during the dry months; and (c) sporadic, when groundwater is not regularly used in the household.

### Health-risk assessment from exposure to ^222^Rn in water

Human exposure to radiation due to ^222^Rn and its short-lived decay products derived from domestic water use was calculated according to the United Nations Scientific Committee on the Effects of Atomic Radiation report (UNSCEAR 2000). Apart from the direct ingestion of water containing ^222^Rn, this approach also includes other domestic water uses that contribute to indoor radon levels and the associated inhalation risk, such as showering. Therefore, estimates of the total effective dose $\left({E}_{\mathrm{total}}\right)$ are divided into ingestion $\left({E}_{\mathrm{ing}}\right)$ and inhalation $\left({E}_{\mathrm{inh}}\right)$, and calculated as below:

1 $${E}_{\mathrm{ing}}=\mathrm{A }{\mathrm{W}}_{\mathrm{ec}} {\mathrm{DCF}}_{\mathrm{ing}}$$

2 $${E}_{\mathrm{inh}}=\mathrm{A }{\mathrm{R}}_{\mathrm{aw}}\mathrm{ F O }{\mathrm{DCF}}_{\mathrm{inh}}$$

where *A* is the ^222^Rn activity in the water supply, ${W}_{\mathrm{ec}}$ is the weighted estimate of water consumption per capita (255.5 L y^−1^), ${\mathrm{DCF}}_{\mathrm{ing}}$ and ${\mathrm{DCF}}_{\mathrm{inh}}$ are the effective dose coefficients applying to ingestion and inhalation, respectively (${\mathrm{DCF}}_{\mathrm{ing}}$=3.5 nSv Bq^−1^ and ${\mathrm{DCF}}_{\mathrm{inh}}$=9 nSv (Bq h^−1^ m^−3^)^−1^), ${R}_{\mathrm{aw}}$ is the water–air transfer coefficient of ^222^Rn indoors (10^−4^), *F* is the equilibrium factor between ^222^Rn and its progeny (0.4) and *O* is the typical indoor occupancy (7000 h y^−1^). ${W}_{\mathrm{ec}}$ is taken from Chen (2019) who compiled cold tap water consumption rates from seven European and North American countries. ${R}_{\mathrm{aw}}$ depends on factors such as indoor volume, ventilation rate, domestic water usage per occupant, and the amount of degassed ^222^Rn during the activities that use water (e.g., showering, clothes-washing, dishwashing; NRC 1999; UNSCEAR 2000). Additionally, ${\mathrm{DCF}}_{\mathrm{ing}}$, ${\mathrm{DCF}}_{\mathrm{inh}}$, *F* and *O* values are taken from the UNSCEAR recommendations for adults (UNSCEAR 1993, 2000).

Excess lifetime cancer risk ($\mathrm{ELCR}$) derived from exposure to ^222^Rn in domestic water was estimated from the calculated total effective dose as follows:

3 $$\mathrm{ELCR}={E}_{\mathrm{total}} \mathrm{LE RF}$$

where $\mathrm{LE}$ is the life expectancy (83.5 years in 2019), obtained from the Instituto Nacional de Estadística (www.ine.es) and $\mathrm{RF}$ is the standard risk estimate per Sv (0.057 Sv^−1^) taken from the ICRP (2005).

## Results and discussion

### ^222^Rn and ^226^Ra in continental waters of the Ría de Vigo basin

**Fig. 3:**
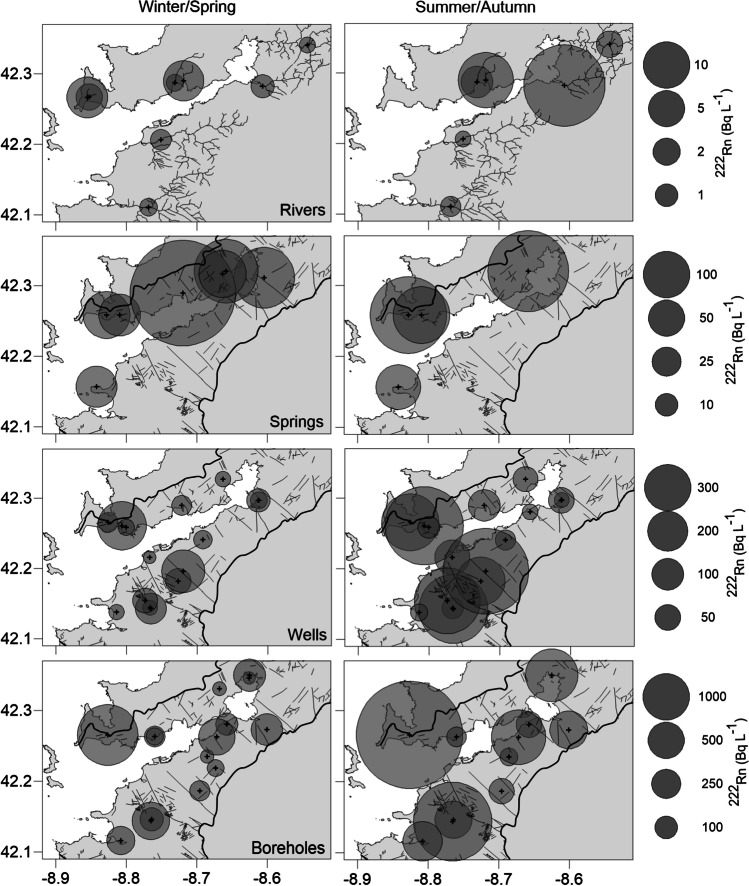
Spatial distribution of ^222^Rn measured in rivers (upper panels), springs, wells, and boreholes (lower panels) from the Ría de Vigo basin during winter/spring (left) and summer/autumn (right). The sampled riverine network is shown in the upper panels for reference, while the basin limits and the main faults and lineaments are shown in the panels containing groundwater data. Note the different scale of ^222^Rn levels used for each horizontal pair of panels

The radon levels in rivers of the Ría de Vigo basin range from 1.2 to 20.2 Bq L^−1^ (average 3.96 Bq L^−1^, *n* = 8; and 7.78 Bq L^−1^, *n* = 6, during the wet winter/spring and the dry summer/autumn seasons, respectively; Fig. 3). In groundwater, ^222^Rn activities are generally one order of magnitude higher than those in surface waters. Wells dug into the regolith exhibit high ^222^Rn activities (average 99.0, *n* = 15 and 241 Bq L^−1^, *n* = 16 during winter/spring and summer/autumn, respectively), comparable to those measured in springs (average 135.5 Bq L^−1^, *n* = 7 and 152.9 Bq L^−1^, *n* = 4 during winter/spring and summer/autumn, respectively). The highest mean ^222^Rn activities are observed in groundwater samples collected from boreholes screened in the fractured rock aquifer layer (average 427 Bq L^−1^, *n* = 14 and 962 Bq L^−1^, *n* = 11 during winter/spring and summer/autumn, respectively).

Radon activities in continental waters of the Ría de Vigo basin were seasonally variable, with summer/autumn levels more than double those measured during winter/spring (*n* = 36; Wilcoxon signed rank test *p* < 0.001; average change: 2.8 ± 0.7 times; Fig. 3). Only 3 of the 36 locations sampled during both periods showed a slight reduction in ^222^Rn activities from winter/spring to summer/autumn. The highest averaged seasonal changes are found in the sampled wells (4.0 ± 1.6 times), followed by rivers (2.7 ± 0.9 times), boreholes (1.9 ± 0.2 times), and springs (1.5 ± 0.1 times).

While none of the surface waters we sampled reached the 100 Bq L^−1^ reference threshold established by European and Spanish legislation, above which remedial actions are recommended (1000 Bq L^−1^ for private water supply outlets), groundwater samples frequently exceeded this threshold. During winter/spring, > 40% of wells and springs and 78% of the boreholes that we sampled had ^222^Rn in water activities above 100 Bq L^−1^. During summer/autumn, this incidence increased to 65% of the wells and springs (*n* = 17) and all boreholes (*n* = 11) that we sampled.

Activities of ^226^Ra in water measured during the summer survey were very low compared to ^222^Rn (see supplementary materials), in line with the low solubility and strong mineral sorption of Ra at low salinities (all measured salinities during summer/autumn < 0.05 except one sample (1.6), see supplementary data; Webster et al. 1995). A comparison of ^226^Ra activities in filtered and unfiltered water samples shows that a significant portion of the measured ^226^Ra is bound to suspended particles, as unfiltered samples showed higher ^226^Ra activities. Even so, bulk ^226^Ra activities never supported more than 7% of the measured ^222^Rn activities.

### Environmental controls of ^222^Rn levels in continental waters of a radon-prone area

**Fig. 4:**
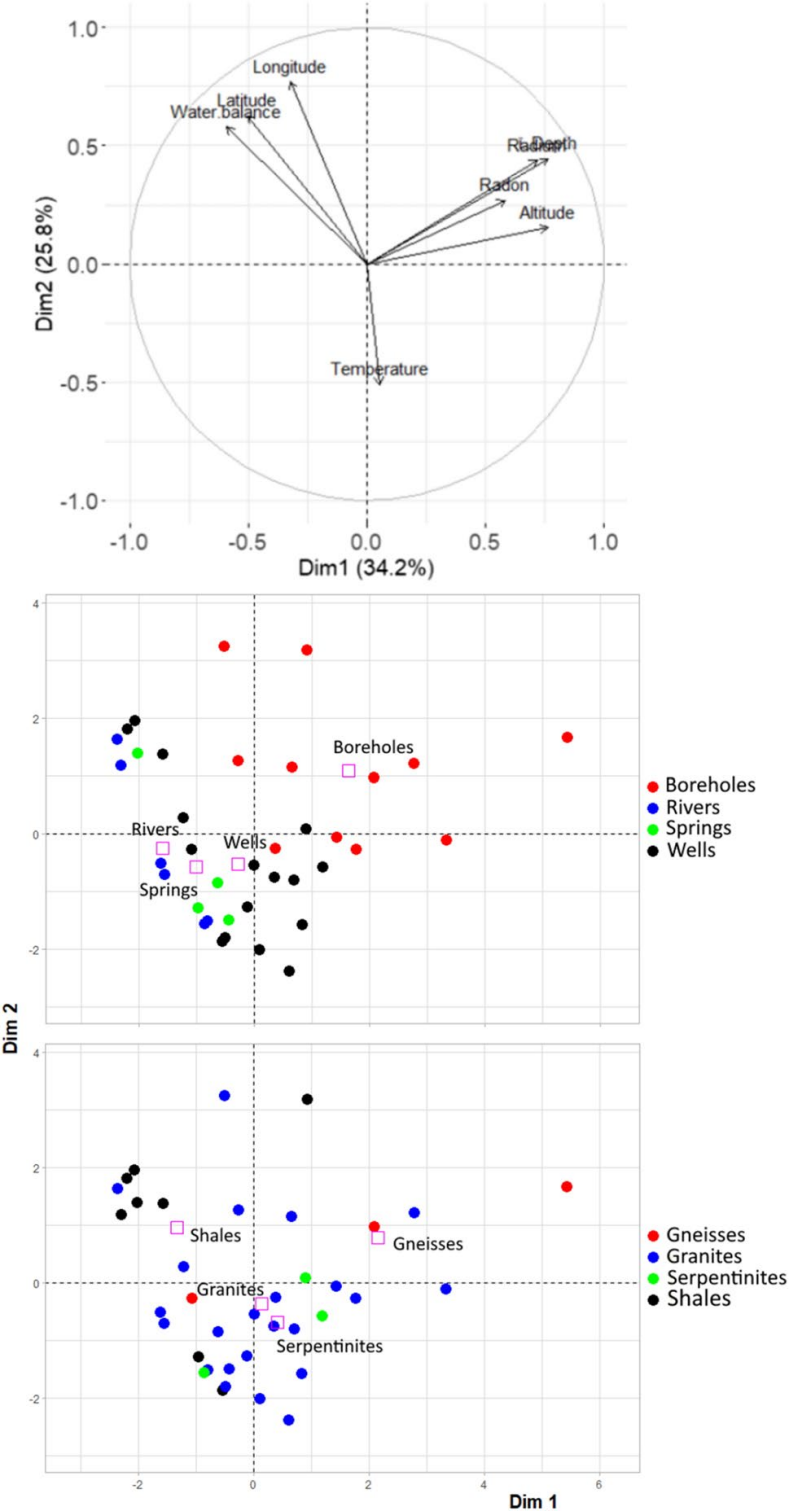
Results of the PCA performed with the data obtained during the summer/autumn survey performed throughout the basin, including scores of the parameters used in the PCA analysis (upper panel) and the results organized by type of water sample (middle panel) and dominant bedrock type (lower panel). Pink squares represent the mean point of each considered group

Together with ancillary and meteorological data, the radioisotope measurements conducted during summer/autumn, i.e., when the highest ^222^Rn activities in water were recorded within the basin, were included in an exploratory principal component analysis (PCA) for our dataset (Fig. 4). The first two components of the PCA explained 60% of the total variability. PCA component 1 links the measured radioisotopes (^222^Rn and ^226^Ra) with depth of well and borehole screening and altitude, while PCA component 2 is governed by location (latitude and longitude) and meteorological variables (water balance and water temperature; Fig. 4). The PCA analysis separated borehole samples from those collected in wells, springs, and rivers, which appeared clustered (Fig. 4). In the case of surface waters, a significant correlation between drainage area and ^222^Rn activities was found both during winter/spring (*r* =  − 0.57; *n* = 8 and *r* =  − 0.77 on a log–log transform) and summer/autumn (*r* =  − 0.47; *n* = 6 and *r* =  − 0.58 on a log–log transform; see supplementary materials).

The influence of local geology (i.e., the differential ^226^Ra content in the basement rocks) over groundwater ^222^Rn levels has been extensively documented (e.g., Banks et al. 1998; Duggal et al. 2020; Sukanya et al. 2021). Nevertheless, no clear influence of local geology was identified in the PCA results (Fig. 4). Instead, during summer/autumn, all the sampled geological units (granites, shales, gneisses, and serpentinites) hosted groundwaters with ^222^Rn activities above the threshold beyond which mitigation action is recommended (see supplementary materials).

The two-layered conceptual aquifer model is instrumental in understanding the major drivers of ^222^Rn activity in Galician groundwaters. Aquifer-specific storage and water transmissivities in the region are two orders of magnitude higher in the highly weathered regolith that feeds wells compared to the fractured rock underneath (Naves et al. 2021; Raposo et al. 2012 and references therein). These characteristics determine contrasting matrix porosity and water content, permeability, groundwater residence times, and hence ^222^Rn emanation rates (Przylibski 2000). Furthermore, ^226^Ra levels in the local aquifer matrix also show contrasting vertical magnitudes in the local aquifers. In soils of the study area, ^226^Ra activity averages 82.2 ± 32.1 Bq kg^−1^ (Quindós et al. 2008) and increases with depth within the regolith (Dios Vidal 1964). Yet, the ^226^Ra activity in local fractured rocks largely exceeds 100 Bq kg^−1^ (Quindós et al. 1994). Higher ^222^Rn emanation rates and water residence times within the fractured rock where the local boreholes are screened and lower specific storage compared to the regolith may thus explain the higher ^222^Rn activity measured in borehole samples. Furthermore, these aquifer units are not homogeneous and thus large differences can be observed within each unit (Fig. 3). Regolith thickness, secondary porosity, spatial and vertical degree of fracturing, the degree of weathering, and ^226^Ra mobilization/precipitation are vertically inhomogeneous in crystalline aquifers (Girault et al. 2018; Przylibski 2000, 2011). These properties affect the aquifer hydraulic properties and ^222^Rn emanation, and may explain the large spatial variability observed within each aquifer unit in the sampled area.

### Drivers of the temporal variability of ^222^Rn in continental waters of the Ría de Vigo basin

**Fig. 5:**
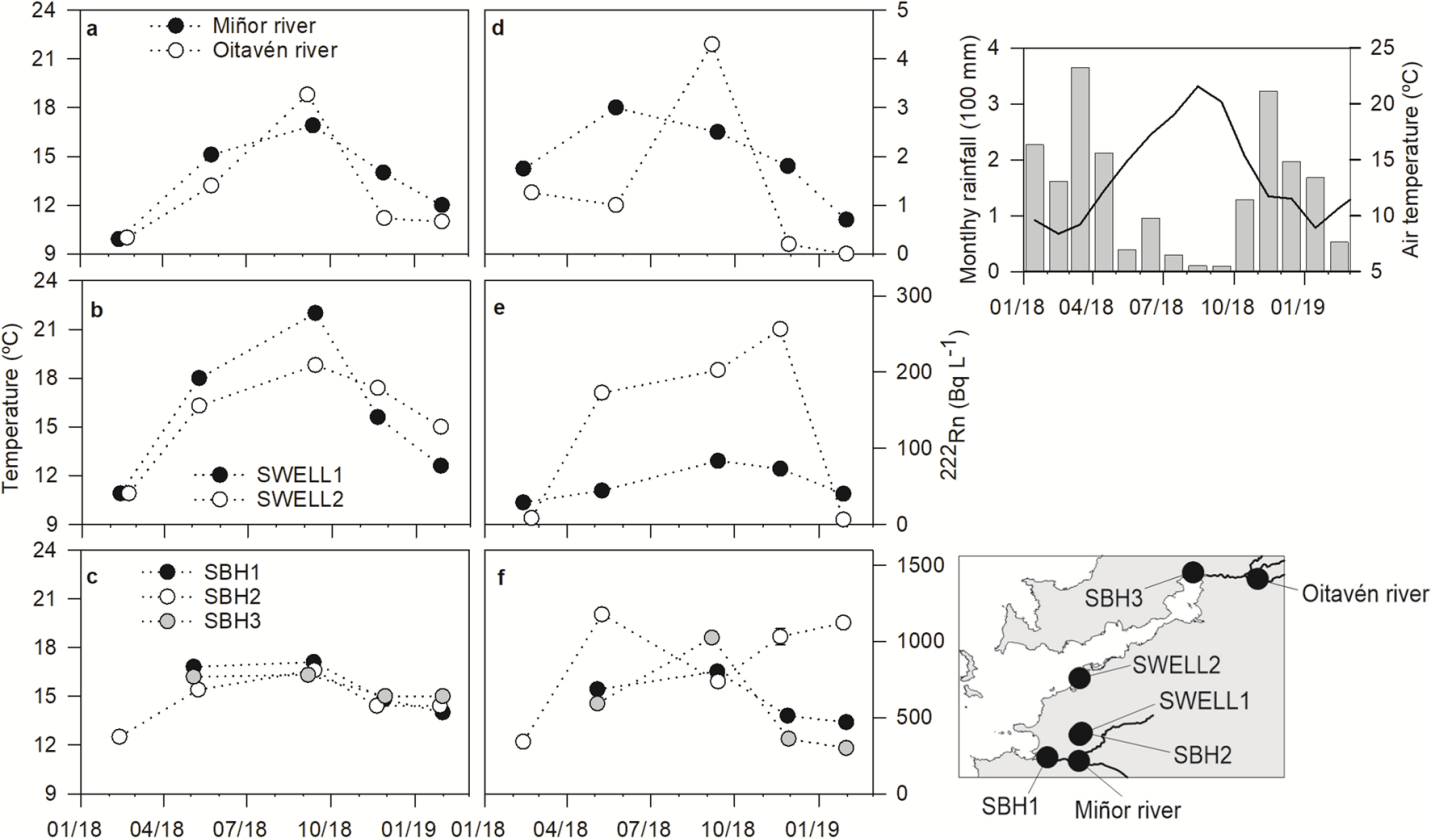
**a**, **b**, **c** Annual variability of water temperature and **d**, **e**, **f** ^222^Rn activities in selected rivers (Miñor and Oitavén), wells (SWELL1 and SWELL2) and boreholes (SBH1, SBH2, and SBH3) from February 2018 to January 2019. **g** Basin-averaged, monthly rainfall (bars), and air temperature (line), obtained from 13 public meteorological stations, are also shown, **h** together with the location of the sampled waters

As observed in the basin-wide surveys, the highest ^222^Rn activities in surface and groundwater were generally measured during summer 2018 and the lowest occurred during winter, following the seasonality of both water temperature and rainfall (Fig. 5). ^222^Rn activities in the Oitavén and Miñor Rivers showed large temporal changes. Particularly notable are the changes registered in the Oitavén River, which is part of the largest river system in the basin and the source of water supply to the biggest municipalities in the region (Fig. 1). There, ^222^Rn activities peaked during the summer and were undetectable during winter 2019. This survey coincided with a large sudden flood, and thus rainfall dilution together with increasing turbulence and therefore ^222^Rn degassing (Rogers 1958) may explain this low value. A significant, negative correlation of ^222^Rn activities and river discharge is observed for both rivers (Oitavén: *r* =  − 0.54; Miñor: *r* =  − 0.83; *n* = 5; see supplementary materials). At low river flows, the relative magnitude of groundwater discharged into the rivers to the overall river flux should increase, while turbulence, and therefore degassing, would be reduced during low water table periods. These factors, in turn, may explain the build-up in ^222^Rn levels during the summer in the local river waters (e.g., Martindale et al. 2016).

The observed seasonal changes were much larger in groundwaters extracted from the regolith (wells) compared to those drawn from the fractured bedrock (boreholes). The faster recharge of the aquifer within the regolith compared to the fractured rock underneath (Raposo et al. 2012) implies that percolation during the wet season would have a more immediate effect on ^222^Rn activities in the surface than deeper groundwater. Furthermore, due to the design of wells (about 1.5–2 m in diameter), larger contact of water with the atmosphere would entail faster groundwater ^222^Rn degassing compared to boreholes. During the dry months, higher water demand would imply shorter water residence times inside the wells and in contact with the atmosphere and could partially explain the observed increase in ^222^Rn activities measured during the summer (Knutsson and Olofsson 2002). In fact, the largest seasonal changes are observed in SWELL2 (Fig. 5), which is almost exclusively used for irrigation purposes. When sampling private wells and boreholes, we pumped water until a constant temperature was reached (typically 10 to 15 min). This ensures that the sample is representative of the water contained inside the groundwater supply unit but is not enough to significantly renew it with water contained inside the aquifer matrix. Thus, ^222^Rn activities recorded during winter in SWELL2 may not be representative of local groundwater ^222^Rn levels due to lack of use, resulting in ^222^Rn evasion and decay from the stagnant well water (e.g., Schubert et al. 2011). On the other hand, the borehole SBH2 showed lower temporal variability or radon activity than the others, and ^222^Rn activity in excess of 600 Bq L^−1^ for most of the sampled period (Fig. 5). Adding to the influence of potential water use on radon levels, these high but stable ^222^Rn activity values suggest lower aquifer recharge and groundwater dynamics compared to other boreholes, highlighting the spatial variability of radon build-up within aquifer units. Water usage may also contribute to the observed seasonal changes in ^222^Rn levels in the remaining private groundwater supply units (see supplementary materials). These changes are also of higher magnitude in wells compared to springs. Nevertheless, springs still show a significant increase in ^222^Rn activities during the summer, and thus other environmental factors may also influence the seasonality observed.

**Fig. 6:**
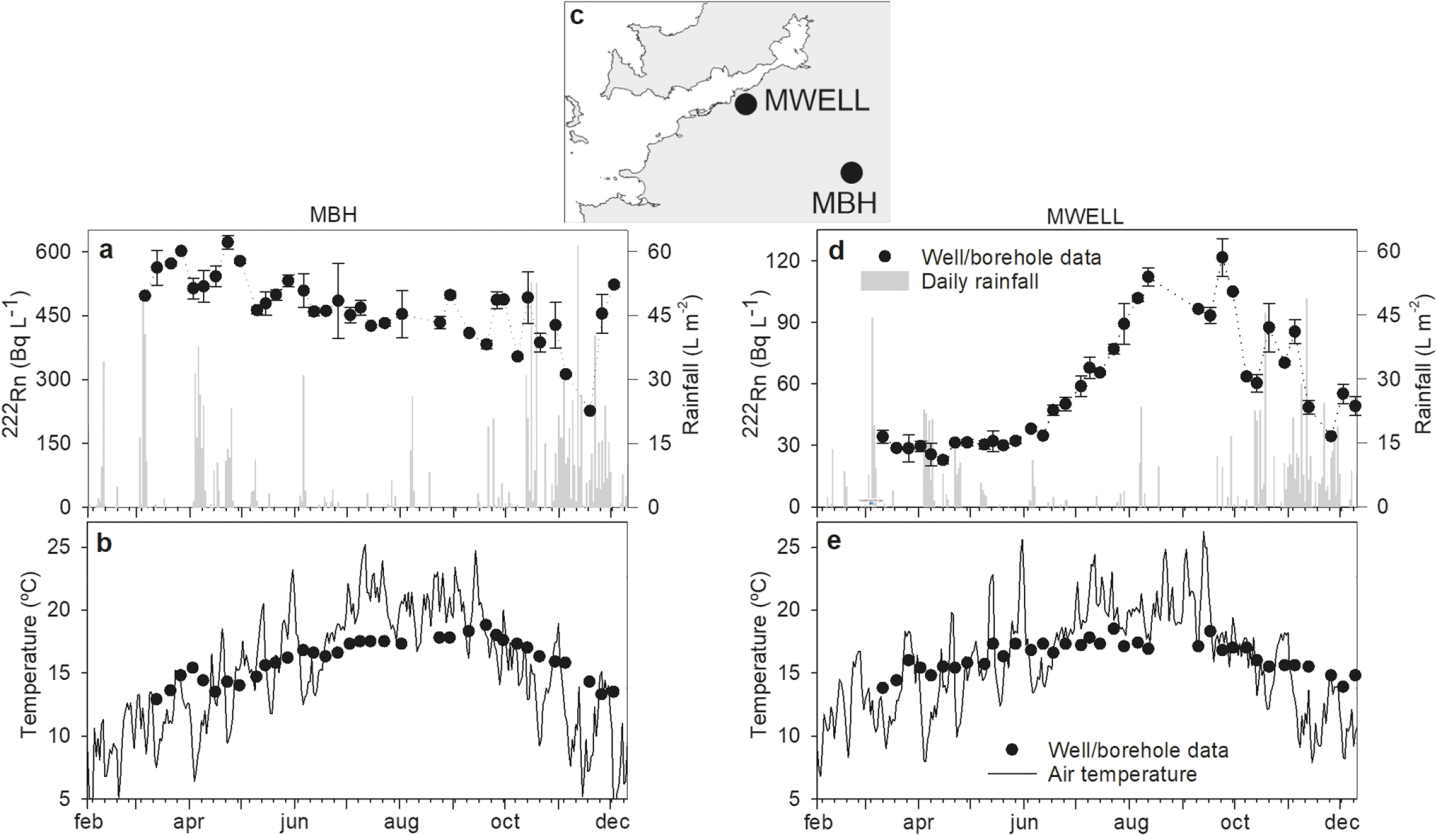
**a**, **d** Radon in water and **b**, **e** water temperature measured in a monitored well (MWELL) and borehole (MBH) on a weekly basis from March to December 2019. **c** Daily rainfall rate and air temperature obtained from the nearest meteorological station of the public network of Meteogalicia (https://www.meteogalicia.gal) are also shown, together with the location of the monitored groundwaters

Strong fluctuations in the measured ^222^Rn activities are observed at different time scales in the two groundwater supply points that were monitored on a weekly basis during 2019 (Fig. 6). A particularly obvious peak in ^222^Rn activities was observed in the absence of rainfall within MWELL, from June to August (Fig. 6). Beyond this peak period, we found significant negative correlations between ^222^Rn activities and weekly accumulated rainfall with a lag time of one week (*r* =  − 0.53, *n* = 12 from March to June and − 0.52, *n* = 14 from August to December). Within borehole MBH (Fig. 6), ^222^Rn activities were higher than 400 Bq L^−1^ for most of the sampled period and were relatively constant with time, even if a sudden drop follows the arrival of autumn rainfall. This borehole is the deepest one sampled (120 m depth), and the stability of ^222^Rn activities, aside from autumn, suggests that groundwater dynamics (specific yield, porosity, recharge, and transmissivity) are of low magnitude there, following the vertical zonation of these properties in crystalline aquifers. There, the relative contribution of the two springs also used in the sampled water supply unit is expected to suddenly increase during rainfall events and would explain the sudden drop in ^222^Rn activities during autumn.

Groundwater degassing (De Francesco et al. 2010), recharge/discharge rates, and water table oscillations are orders of magnitude higher in the regolith compared to the fractured rock underneath in the study region (Naves et al. 2021; Raposo et al. 2012). Together with the low atmospheric ^222^Rn activity with respect to groundwaters characteristic of the study area (< 20 Bq m^−3^; Ibánhez et al. 2021), these factors explain the dilution effect on ^222^Rn activities in the regolith brought by aquifer recharge with rainfall. In contrast, De Francesco et al. (2010) found the opposite effect of rainfall over groundwater ^222^Rn content in a Mediterranean aquifer system dominated by pyroclastic and alluvial deposits in contact with carbonate reservoirs. They noticed that ^222^Rn levels increased particularly during the first rains after the dry season and attributed it to a downward transport of ^222^Rn during rainfall percolation that became particularly acute when the water table was at its annual lowest. In aquifers of simple lithology, ^222^Rn emanation from the reservoir rock commonly peaks at the surface and decreases with depth (e.g., Sukanya et al. 2021). However, this is not the case in the study area, with ^222^Rn levels over an order of magnitude higher in boreholes compared to the sampled wells. Relatively low ^222^Rn emanation rates in the regolith together with the fast infiltration observed (about a week) may explain the prevailing diluting effect of rainfall recharge on groundwater ^222^Rn levels observed in the Ría de Vigo basin.

Although rainfall recharge dilutes ^222^Rn levels in the regolith during the wet season, it may not be enough to explain the large increase in ^222^Rn levels during the dry season observed in almost all the sampled groundwaters. The increase in ^222^Rn due to aquifer matrix ^222^Rn emanation after cessation of rainfall dilution would be faster (99% of the maximum supported ^222^Rn activity reached in 24 days; Przylibski 2000) than the observed build-up of ^222^Rn (sustained increase for more than 60 days, from June to August, observed in MWELL; Fig. 6). Larger groundwater retention times (and therefore larger contact times with the aquifer rock; e.g., Hoehn and Gunten 1989) would contribute to the observed increase of ^222^Rn activity during the dry months. Additionally, density instabilities caused by vertical temperature gradients can also explain daily to seasonal ^222^Rn changes in groundwaters (Choubey et al. 2011; Kamra 2015). The seasonal fluctuation of air temperature drives groundwater temperature changes and this, in turn, promotes inverse temperature gradients within the aquifer that could trigger ^222^Rn transport from the deeper layers to the surface by convective overturn. Data from the well SWELL1 and borehole SBH2 that lay next to each other (Fig. 5) show this seasonal thermal inversion: during both sampled winters, groundwaters from SBH2 were 1.8 and 1.6 °C warmer than those from SWELL1. However, during the spring, summer, and autumn, the temperature gradient reversed, peaking during the summer with a temperature difference of 5.4 °C between both aquifer layers. Similarly, this is observed in the weekly sampled well MWELL, where the unsupported ^222^Rn build-up almost perfectly matches the period where daily averaged air temperatures became higher than groundwater temperatures (Fig. 6).

The non-dimensional Rayleigh number allows the determination of thresholds for the onset and intensity of convection in porous media (e.g., Rocha 2000):

4 $${R}_{a}=\frac{\alpha \Delta T \gamma g H}{v k}$$

where α is the thermal expansion coefficient, $\Delta T$ is the temperature gradient observed, $\gamma$ is the permeability, *g* is the gravitational force, *H* is the depth scale, $v$ is the kinematic viscosity, and *k* is the ^222^Rn diffusivity corrected for tortuosity. Using tabulated and literature values, including those determined in Galician soils (Raposo et al. 2012; Schubert and Paschke 2015 and references therein), a temperature gradient of 0.05 °C m^−1^ would give a *R*_*a*_ number higher than the critical value for free convection to develop (4π^2^), a threshold that is seasonally surpassed in the study area. This has strong implications for human exposure to ^222^Rn from groundwaters due to the large vertical gradient in ^222^Rn content observed in the study area; groundwaters from the regolith can, in fact, contain much larger ^222^Rn activities than those supported by bedrock emanation, transporting ^222^Rn-rich waters from depth. Together with the current predictions of air temperature rise, a decrease in annual recharge, and a concentration of rainfall during winter and spring due to climate change, thus prolonging the duration of the dry season (Raposo et al. 2013), imply that an increase in summer ^222^Rn content in groundwater within the regolith can be expected. These factors would also imply an increase in water extraction from groundwater resources, contributing to an increase in ^222^Rn content in the extracted groundwaters by limiting groundwater degassing and ^222^Rn decay, and therefore increasing human exposure to water-borne radiation.

### Risk assessment of exposure to ^222^Rn in continental waters of a radon-prone area

Annual individual radiation dose follows that of the ^222^Rn activities found in the different water sources, with highest average radiation doses received from the exclusive use of borehole water and the lowest from the use of river water (Table 1). In all cases, the largest radiation dose comes from inhalation (Hopke et al. 2000; Table 1), accounting for more than 70% of the total effective dose received.

**Table 1:** Calculated ingestion (${E}_{\mathrm{ing}}$), inhalation (${E}_{\mathrm{inh}}$) and total effective doses (${E}_{\mathrm{total}}$) together with estimated lifetime cancer risk ($\mathrm{ELCR}$) derived from measured ^222^Rn in continental water samples from the Ría de Vigo basin. ${E}_{\mathrm{ing}}$ quantifies the radiation dose from the direct ingestion of water containing ^222^Rn, while ${E}_{\mathrm{inh}}$ quantifies the radiation dose to adults associated with the contribution of other water domestic water uses that promote water degassing and thus contribute to indoor ^222^Rn levels. *n* denotes the number of independent observations, and the minimum and maximum values are presented in parentheses. Only data sampled during the two basin-wide surveys is used

Origin of water supply	*n*	Mean depth of screening	${E}_{\mathrm{ing}}$	${E}_{\mathrm{inh}}$	${E}_{\mathrm{total}}$	$\mathrm{ELCR}$
		(m)	(µSv y^−1^)	(µSv y^−1^)	(µSv y^−1^)	10^−2^
Rivers	6	-	4.9 ± 1.6 (1.7–10.5)	13.8 ± 4.6 (0.4–2.5)	18.6 ± 6.2 (6.6–40.1)	0.009 ± 0.003 (0.003–0.019)
Springs	4	-	127 ± 20 (83–178)	357 ± 56 (233–502)	484 ± 76 (316–680)	0.23 ± 0.04 (0.15–0.32)
Wells	15	11.3 (8–17)	159 ± 34 (33–408)	447 ± 94 (94–1150)	606 ± 128 (128–1558)	0.29 ± 0.06 (0.06–0.74)
Boreholes	11	44.5 (28–80)	663 ± 153 (104–1858)	1869 ± 432 (292–5234)	2532 ± 585 (396–7092)	1.21 ± 0.28 (0.19–3.38)

The World Health Organization (WHO) has set a maximum recommended radiation exposure of 0.1 mSv y^−1^ from a single source for an adult. The annual radiation doses estimated to result from ^222^Rn ingestion are lower than this threshold when river water is the sole untreated source of domestic water. Due to the low ^226^Ra content in the sampled waters and its long half-life, once degassed, ^222^Rn ingrowth from its parent isotope can be assumed negligible during ingestion. Nevertheless, the reliance on groundwater extracted from half of the sampled wells (*n* = 16), 3 out of 4 sampled springs, and all the sampled boreholes (*n* = 14) would result in ^222^Rn ingestion radiation exposure exceeding the WHO’s recommended dose limit (0.1 mSv y^−1^). Furthermore, when considering the total radiation dose estimated from both ingestion and inhalation of ^222^Rn derived from domestic water usage, the WHO’s safety threshold for individual radiation exposure would only be cleared if river water were the sole source of domestic water supply. All the groundwater samples (springs, wells, boreholes) represent sources of domestic water that would result in total effective radiation doses associated with ^222^Rn higher than 0.1 mSv y^−1^, up to a maximum of 5.7 mSv y^−1^ (Table 1). This highlights the high risk associated with reliance on untreated groundwater for domestic use in the study area.

**Fig. 7:**
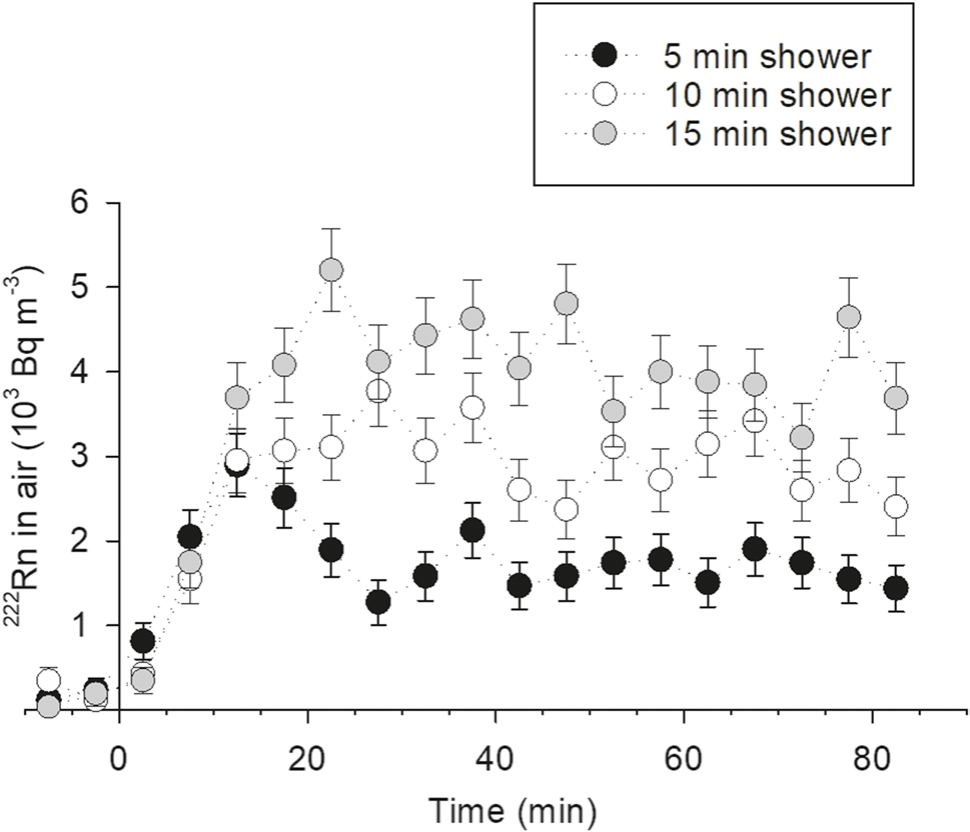
Build-up of ^222^Rn in air in the sampled bathroom (1.4 × 2.6 × 2.3 m) during shower operation (i.e., water running through the shower head) lasting 5, 10, and 15 min. Water is sourced from a semi-public borehole with a ^222^Rn content of 592 ± 11 10^3^ Bq m^−3^ and a temperature of 16.0 °C measured at the time of the experiments. Time is counted from the moment the taps are turned and waters start flowing through the shower head

Inhalation risks associated with ^222^Rn in water used for domestic purposes come from indoor water degassing due to daily activities such as doing the laundry or showering. The activity of ^222^Rn in groundwater sourced from the borehole supplying the shower used to measure ^222^Rn levels inside the bathroom was 592 ± 11 Bq L^−1^ during the experiments. Once the shower was turned on, the ^222^Rn activities inside the bathroom rapidly increased (initial ^222^Rn activities inside the bathroom were 116, 346, and 37 Bq m^−3^ prior to water flow for 5, 10, and 15 min, respectively; Fig. 7) and continued to increase if the water was flowing. Once a peak was reached, the radon in air rapidly mixed throughout the bathroom space and reached a high plateau that depended on how long the shower was on for (1814 ± 26, 2982 ± 27, and 4149 ± 38 Bq m^−3^ in air after 5, 10, and 15 min of water flow through the shower head, respectively). These data show that a large proportion of the ^222^Rn dissolved in water outgassed during shower use (70, 58, and 54% of degassing after 5, 10, and 15 min of water flow, respectively). Since the water used in our shower experiments was not heated, these degassing rates can be considered minimum values, as they are expected to increase with increasing water temperature (Schubert and Paschke 2015).

While our inhalation radiation dose estimate includes that from ^222^Rn and its short-lived ^222^Rn decay products under the assumption of equilibrium, the interpretation in the context of health risk associated with acute and short-lived exposure demand extra caution. The ingrowth of short-lived ^222^Rn decay products from the release of ^222^Rn is estimated to take at least half an hour (NRC 1999). Thus, during a short shower, direct exposure to these short-lived ^222^Rn decay products is likely to be low and dependent on how long the person remains in the shower room. Nevertheless, if the room is not ventilated at sub-hourly timescales, the ^222^Rn released by degassing of shower water will contribute to the individual’s domestic exposure to ionizing radiation by increasing overall indoor ^222^Rn levels. As observed elsewhere, lifetime lung cancer risk in the study area more than doubles when residential ^222^Rn levels are above 200 Bq m^−3^ compared to individuals exposed to chronic residential ^222^Rn levels lower than 100 Bq m^−3^ (Torres-Durán et al., 2014). Residential degassing of ^222^Rn-rich groundwaters adds to the widespread high indoor ^222^Rn levels characteristic of the study area (e.g., Barros-Dios et al. 2007; López-Abente et al. 2018) and highlights the health risks associated with the domestic use of untreated ^222^Rn-rich waters.

Whole-body radiation dose associated with domestic use of groundwater in the study area may indicate an increased risk of developing cancer over a lifetime. Calculated excess lifetime cancer risk associated with the long-term use of untreated groundwaters for domestic water supply was on average 5.73 ± 1.23 × 10^−3^ (i.e., 0.57 ± 0.12%; Table 1), almost 20 times higher than the worldwide average (0.29 × 10^−3^; UNSCEAR 2000). In a pioneering study performed in the autonomous region of Galicia, Pérez-Ríos et al. (2010) attributed 3 to 5% of lung cancer mortality to ^222^Rn exposure, which increased up to 22%, when combined with the effects of smoking. Recently, Ruano-Ravina et al. (2021) raised this value to 7.0%, the highest lung cancer mortality attributable to ^222^Rn exposure in Spain. Furthermore, López-Abente et al. (2018) found epidemiological evidence for the association of indoor ^222^Rn levels with lung, stomach, and brain cancer mortality in Galicia. They reported a mortality increase of 9, 17, and 28% caused by lung, stomach, and brain cancer, respectively, associated with a two-fold increase in ^222^Rn exposure. Although inhalation is the largest contributor to the total effective dose estimates associated with the domestic use of water calculated here, ^222^Rn ingestion doses derived from the consumption of untreated groundwaters from the region were, on average, higher than the maximum radiation exposure recommended by the WHO. Thus, human exposure to ^222^Rn via contribution to indoor levels but also via ingestion represents a significant health risk that requires specific attention in the study area.

## Conclusions

The characteristic hydrogeology of crystalline basins provides insight into the dynamics of ^222^Rn in the continental waters of the Ría de Vigo basin. The much lower ^222^Rn activities and higher temporal variability observed in wells compared to boreholes can be explained by orders of magnitude higher specific storage, transmissivity, and recharge rates, as well as lower ^222^Rn emanation from the surface regolith compared to the underlying fractured rock units. Additionally, convective overturn of groundwater driven by thermal gradient reversal can bring ^222^Rn-rich waters from the fractured rock up to the regolith, potentially contributing to the build-up of ^222^Rn activities observed in the sampled wells during the dry season. This mechanism of ^222^Rn enrichment of surface aquifer layers could be enhanced if current projections of climate change for the region are verified. Incidentally, these would also lead to increased reliance on groundwater for domestic water supply due to expected lowering of surface water reserves. The combination of the two factors would amplify the risk of exposure to ^222^Rn-in-water and therefore of detrimental health impacts on the local population.

All groundwater samples analyzed in this study, if used untreated as the sole source of water for domestic purposes, would result in total effective radiation doses exceeding the limit recommended by the WHO for adult individuals. Considering the widespread occurrence of high ^222^Rn levels, the high proportion of the local population that relies solely on groundwater supply and the high radiation exposure arising from domestic water usage, remedial actions in groundwater supply units are recommended for radiological protection of the local population. The largest contribution to the total effective radiation dose from ^222^Rn in water comes from inhalation, with lower contributions coming from ingestion. Thus, remedial actions aiming at limiting human exposure to ^222^Rn in water should be implemented before the water is drawn into dwellings to prevent ^222^Rn degassing indoors.

## Supplementary Information

Below is the link to the electronic supplementary material.

Supplementary file1 (DOCX 918 KB)

Supplementary file2 (XLSX 21 KB)

## Data Availability

The basin-wide data used in this study can be found in supplementary materials. The remaining data will be made available in the Digital CSIC open repository upon acceptance for publication.
