# Supplementary material for: Radon prevalence in domestic water in the Ría de Vigo coastal basin (NW Iberian Peninsula)
Source: Environ Sci Pollut Res Int. 2023 May 4;30(27):69927–40. doi: 10.1007/s11356-023-27305-6 (PMC10239377; doi:10.1007/s11356-023-27305-6)
Supplement: Supplementary file 1 — Supplementary file1 (DOCX 918 KB) [file 11356_2023_27305_MOESM1_ESM.docx]

**Supplementary Materials of Manuscript**: Assessment and analysis of Radon prevalence in domestic water in the Ría de Vigo coastal basin (NW Iberian Peninsula)

J. Severino P. Ibánhez^1,2^*, Xosé Antón Álvarez-Salgado^1^ and Carlos Rocha^2^

^1^Laboratorio de Geoquímica Orgánica, Instituto de Investigacións Mariñas (IIM), Consejo Superior de Investigaciones Científicas (CSIC), Vigo, SPAIN

^2^Biogeochemistry Research Group, School of Natural Sciences, Trinity College Dublin, Dublin, IRELAND

*Corresponding Author: J.S.P. Ibánhez, [jseverino@iim.csic.es](mailto:jseverino@iim.csic.es)

Content:

Supplementary Figure 1.

Supplementary Figure 2.

Supplementary Figure 3.

Supplementary Figure 4.

Supplementary Figure 5.


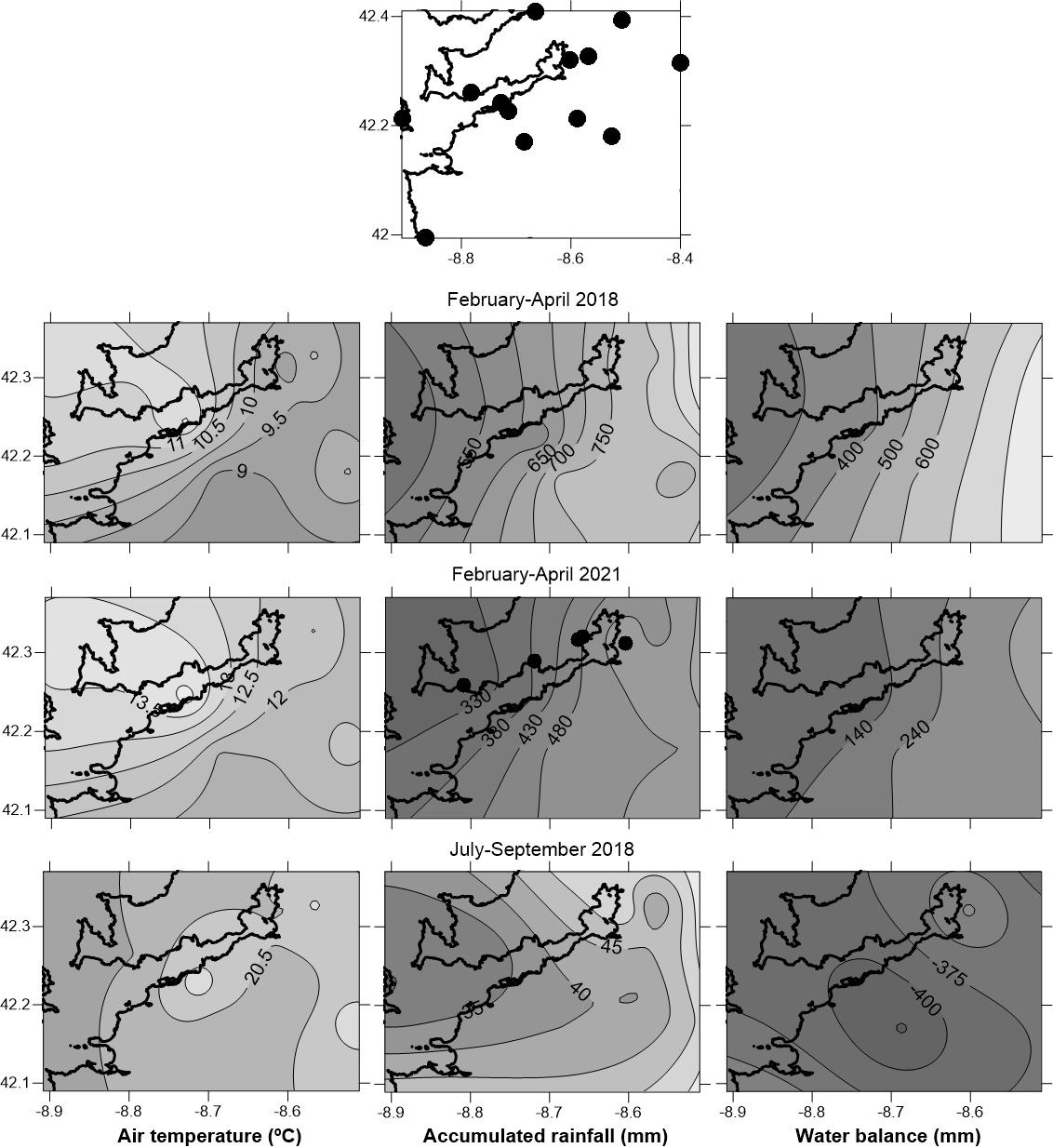


Supplementary Figure 1. Air temperature, accumulated rainfall and water balance in the study area during February-April 2018, February-April 2021 and July-September 2018. The data is interpolated from that of the 13 public meteorological stations in the area (see the location of the meteorological stations in the upper panel).


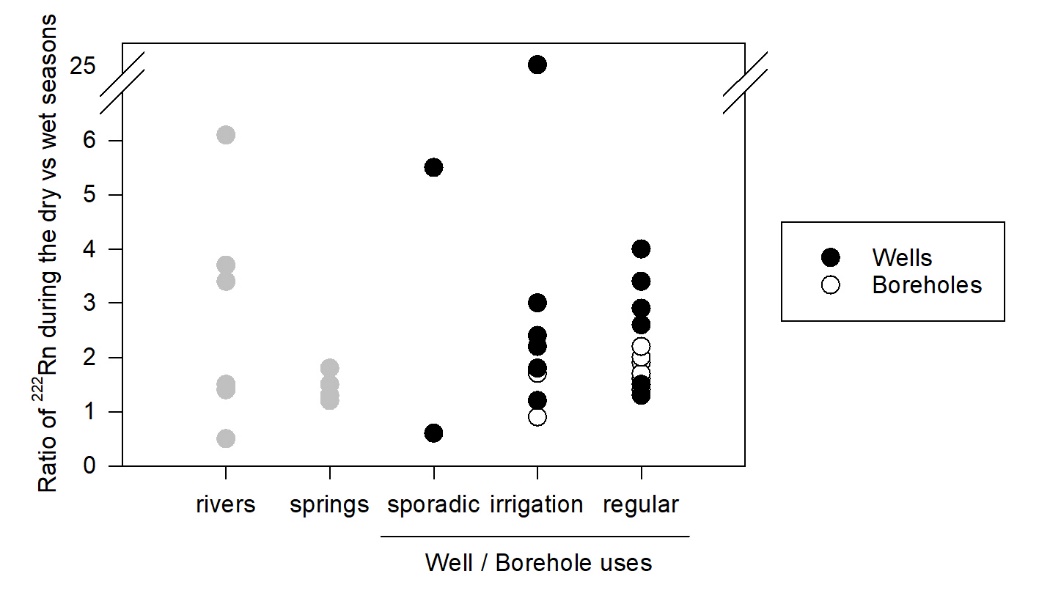


Supplementary Figure 2. Ratio of ^222^Rn activities measured during the dry and wet seasons in continental waters of the Ría de Vigo basin. Private groundwater supply units are organized by use: sporadic (no sustained use), irrigation (large seasonal difference in water use) and regular (similar water use throughout the year).


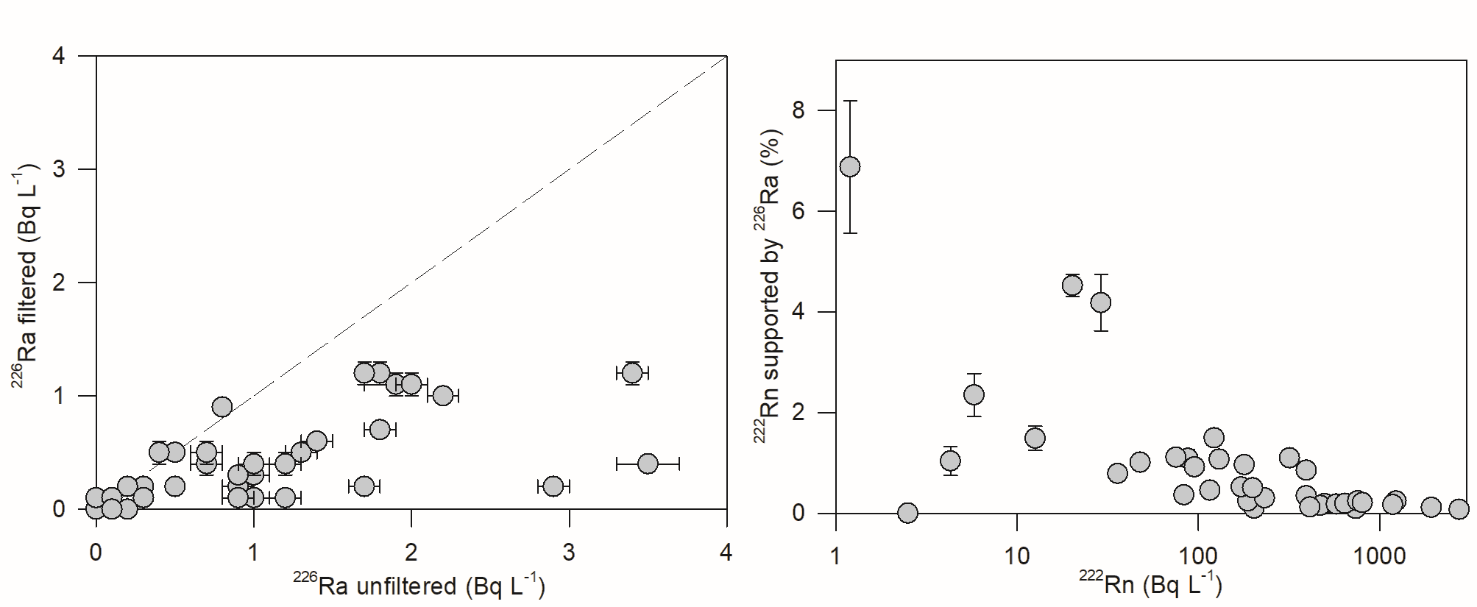


Supplementary Figure 3. Relationship between ^226^Ra activities measured from the ingrowth of ^222^Rn in water collected during the summer/autumn basin-wide survey for unfiltered and filtered samples (left; note the 1:1 dashed line for reference). The proportion of ^222^Rn supported by its parent isotope ^226^Ra is shown as a function of measured ^222^Rn activities (note the logarithmic scale of the x axis).


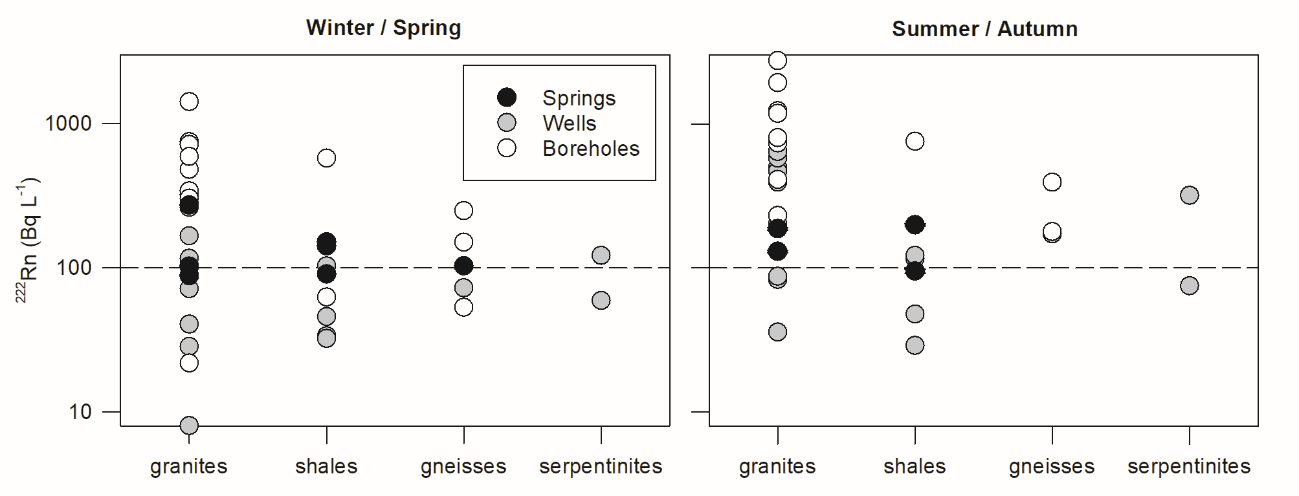


Supplementary Figure 4. Measured ^222^Rn activities organized by dominant bedrock type during winter-spring and summer-autumn (note the logarithmic scale).


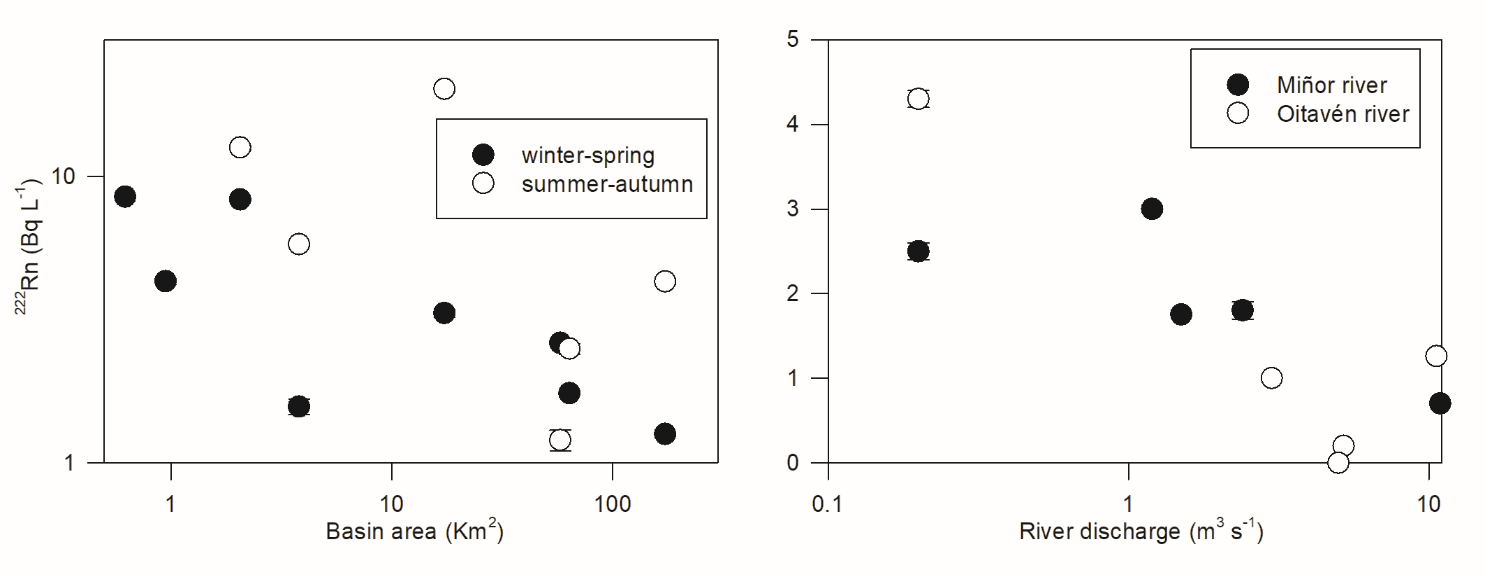


Supplementary Figure 5. Relationships between ^222^Rn activities in the sampled rivers and river basin area (left panel, note the logarithmic scale) and that of ^222^Rn activities in the seasonally sampled rivers (Oitavén and Miñor rivers) and river discharge (right panel.
